# Spatial Suitability of Peste des Petits Ruminants in North Africa Using Machine-Learning Ecological Niche Modeling

**DOI:** 10.3390/pathogens15050466

**Published:** 2026-04-24

**Authors:** Dinara Imanbayeva, Moh A. Alkhamis, John M. Humphreys, Andres M. Perez

**Affiliations:** 1 Center for Animal Health and Food Safety (CAHFS), College of Veterinary Medicine, University of Minnesota, Saint Paul, MN 55108, USA; 2 Department of Epidemiology and Biostatistics, Faculty of Public Health, Health Sciences Centre, Kuwait University, Kuwait City 13060, Kuwait; 3 Agricultural Research Service, National Bio and Agro-Defense Facility, U.S. Department of Agriculture, Manhattan, KS 66502, USA; 4 Department of Veterinary Population Medicine, College of Veterinary Medicine, University of Minnesota, Saint Paul, MN 55455, USA

**Keywords:** peste des petits ruminants, PPR, ecological niche modeling, machine-learning, spatial suitability, Random Forest, extreme gradient boosting, support vector machine, North Africa, spatial cross-validation

## Abstract

Peste des Petits Ruminants (PPR) is a highly contagious viral disease of small ruminants and remains a major threat to food security and rural livelihoods across Africa, the Middle East, and Asia. In the Mediterranean, uneven outbreak reporting and intense spatial clustering hinder the identification of regions where environmental and anthropogenic conditions favor disease occurrence. This study applied an interpretable machine-learning ecological niche modeling framework to characterize PPR spatial suitability in North Africa. A merged outbreak dataset (*n* = 744) was compiled from the Food and Agriculture Organization (FAO) EMPRES-i and the World Animal Health Information System (WAHIS) databases for 2005–2026. Outbreak locations were linked to environmental and anthropogenic predictors, spatially thinned, and paired with randomly sampled pseudo-absences at a 1:1 ratio. After correlation-based screening and Boruta feature selection, four classifiers were compared under five-fold spatial block cross-validation: a generalized linear model (GLM), a support vector machine (SVM), Random Forest (RF), and extreme gradient boosting (XGBoost). All models showed good discriminatory performance. Random Forest (RF) and extreme gradient boosting (XGBoost) yielded the highest area under the receiver operating characteristic curve value (AUC = 0.94). Random Forest achieved the highest specificity, XGBoost achieved the highest sensitivity, and the support vector machine showed the most even sensitivity–specificity tradeoff among the machine-learning classifiers. Sheep density, mean diurnal temperature range, temperature seasonality, and human population density were consistently the dominant drivers. Predicted PPR suitability based on reported outbreaks was concentrated along the North African coastal belt and low across most arid inland regions. These findings suggest that passive surveillance is likely to be most informative in coastal production systems where host density, environmental suitability, and reporting opportunity overlap. At the same time, areas of lower reported-outbreak suitability should not be interpreted as disease-free and may require complementary active surveillance approaches.

## 1. Introduction

Peste des Petits Ruminants (PPR) is a high-priority disease for the Global Framework for the Progressive Control of Transboundary Animal Diseases (GF-TADs) as it is a highly contagious and often fatal viral disease that affects small ruminants. PPR severely threatens food security and rural livelihoods in 70 countries across Africa, the Middle East, and Asia [1,2,3,4]. PPR is a Transboundary Animal Disease (TAD) that imposes substantial socio-economic burdens on affected countries through high morbidity and mortality of livestock populations [5]. The effect of the disease on rural communities’ livelihoods can be devastating, with annual losses estimated at USD 1.5 to 2 billion across more than 70 affected countries in Africa, the Middle East, and parts of Asia [4]. With morbidity rates reaching up to 90% and mortality rates as high as 70%, PPR has devastating socio-economic impacts on pastoralist and agrarian communities [3]. PPR is caused by Peste des Petits Ruminants virus (PPRV), a member of the species *Small ruminant morbillivirus* within the genus *Morbillivirus*, subfamily *Orthoparamyxovirinae*, family *Paramyxoviridae* [6]. PPRV is an enveloped virus with a single-stranded, negative-sense RNA genome [7,8]. Four distinct PPRV lineages (I–IV) are distinguishable via sequence variations in a partial N gene region [8].

Historically, lineages I–II have been primarily detected in West Africa, whereas lineage III has circulated in the Middle East and Eastern Africa. After its likely origin in Western Africa, lineage IV spread eastward and became the predominant lineage across Asia, before re-emerging in Africa, where it now appears to be the most prevalent lineage [8,9,10]. North Africa is a key epidemiological crossroads for PPR, bordering the Sahel and embedded in extensive Mediterranean livestock trade networks [11]. The region has a critical interface between endemic and historically free areas, where intense animal movement and environmental gradients shape transmission potential [12,13,14]. A regional review of Transboundary Animal Diseases in North Africa highlighted that vulnerability is amplified by illegal animal movement, communal clashes/conflict, under-reporting of outbreaks, and poor vaccination coverage, alongside broader constraints on national veterinary service capacity and budgets. These are conditions that collectively sustain (re)introductions and silent spread [13]. Also, the recurrent emergence of PPR outbreaks in North Africa has been framed as a non-trivial incursion risk for European Union countries, underscoring that North African circulation can have consequences beyond the region [15]. While effective live-attenuated vaccines exist, eradication programs depend on vaccination strategies aligned with local epidemiology and coordinated between neighboring countries to limit transboundary spread, supported by strengthened surveillance and post-vaccination monitoring [1,2,4,14]. However, conventional surveillance and risk-assessment approaches may be less informative in settings characterized by heterogeneous reporting, complex transboundary dynamics, and non-linear ecological relationships.

In recent years, machine-learning (ML)-based spatial modeling has increasingly been used to integrate outbreak occurrence with environmental and anthropogenic predictors to improve predictive performance and generate actionable insights for transboundary diseases, including PPR. For example, ML approaches have been applied to foot-and-mouth disease risk mapping using meteorological covariates to support spatial risk prediction and test hypotheses about pathogen spread pathways [16]. Despite strong predictive performance, uptake of ML in animal health decision-making is often constrained by the perceived “black-box” nature of these models [16]. Interpretable ML frameworks (SHAP for feature attribution, ICE plots for instance-level visualization) mitigate this black-box limitation [17,18]. Embedding interpretability within PPR risk mapping strengthens inference, improves risk communication, and better aligns model outputs with surveillance planning.

To address the gaps, we applied an interpretable machine-learning ecological niche modeling workflow to characterize PPR spatial suitability in North Africa, using outbreak reports compiled from a broader Mediterranean-interface domain and linked environmental and anthropogenic predictors. The study had three aims: (i) to evaluate multiple machine-learning classifiers for PPR suitability mapping in North Africa, including a generalized linear model (GLM), Random Forest (RF), a support vector machine (SVM), and extreme gradient boosting (XGBoost), using correlation filtering, Boruta feature selection, and spatial cross-validation [19,20,21,22,23,24,25,26,27,28], and identify the best-performing model; (ii) to identify the primary predictors and interaction patterns shaping mapped suitability and interpret their ecological and epidemiological meaning; and (iii) to translate these results into map-based outputs that can support surveillance prioritization and risk-based control planning in North Africa [29].

## 2. Materials and Methods

This study applied a machine-learning ecological niche modeling workflow adapted from Alkhamis et al. [16] to characterize PPR spatial suitability in North Africa. The analysis combined merged outbreak records, environmental and anthropogenic raster predictors, pseudo-absence sampling points, predictor screening, and four classification algorithms evaluated under five-fold spatial block cross-validation. After model performance was compared across algorithms, variable-importance measures, centered individual conditional expectation plots, interaction diagnostics, and Shapley-value explanations were used to examine predictor effects, non-linear responses, and local feature contributions, with the main ecological interpretation focused on the retained model [16,30].

### 2.1. Outbreak Data and Study Region

**Figure 1:**
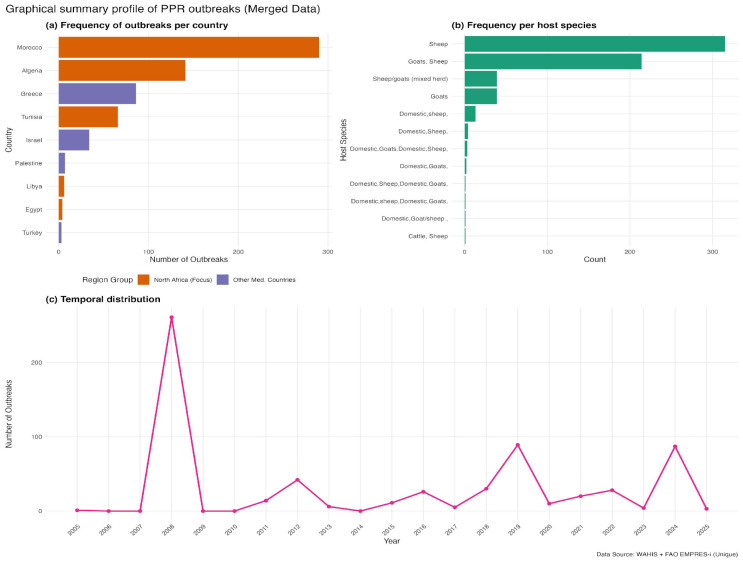
Graphical summary of reported Peste des Petits Ruminants (PPR) outbreaks in North Africa and the broader Mediterranean-interface calibration domain, 2005–2026, based on merged FAO EMPRES-i and WOAH WAHIS data. (**a**) Frequency of reported outbreaks by country. (**b**) Frequency of reported outbreaks by host species category. (**c**) Annual temporal distribution of reported outbreaks.

We compiled PPR outbreak records from two international reporting systems, the Food and Agriculture Organization (FAO) EMPRES-I platform (https://empres-i.apps.fao.org/ (accessed on 12 February 2026)) and the World Animal Health Information System (WAHIS) interface (https://wahis.woah.org/ (accessed on 12 February 2026)), to reduce the likelihood of missing events captured by only one source. EMPRES-i contributed 660 records and the WAHIS had 630 validated outbreak reports. The temporal distribution of compiled reports and the spatial contribution of EMPRES-i and WAHIS are shown in Supplementary Figure S1. Records from the two systems were compared using a spatiotemporal matching rule of ≤10 km and ≤14 days. These thresholds were used to account for differences in geolocation precision and reporting dates across global databases, where coordinates may represent administrative centroids and notification dates may not coincide exactly with outbreak onset. After harmonization, duplicate removal, and spatial thinning, 744 outbreak locations were retained in the merged global dataset (Supplementary Figure S1). For the North Africa modeling domain, duplicate records with identical coordinates were removed after merging, and the remaining points were spatially thinned using a minimum nearest-neighbor distance of 10 km. After thinning, 359 unique PPR-positive locations were retained for ecological niche modeling, of which 352 remained in the final modeling dataset after raster extraction and complete-case filtering. Of these 352 retained PPR-positive locations, 246 were reported in both EMPRES-i and WAHIS, 57 were unique to EMPRES-i, and 49 were unique to WAHIS. The merged analytic dataset retained source identifiers, country, event date, coordinates, and host-category information for each outbreak record prior to deduplication and thinning. Before outbreak extraction, we manually defined and screened a 22-country Mediterranean-interface domain to establish a consistent regional boundary for data assembly and model development. This approach avoided delineating the study area based on the observed outbreak pattern and retained a broader regional extent that captured the environmental and anthropogenic gradients surrounding North Africa. Within this predefined domain, each country was assessed for reported PPR outbreaks during the study period, and the final dataset included only those countries contributing eligible records. This broader Mediterranean-interface domain informed model fitting by capturing a wider range of relevant gradients, whereas all suitability mapping and interpretation were restricted to North Africa, the predefined geographic focus of the study. Outbreaks were identified in 16 countries: Albania, Algeria, Bulgaria, Croatia, Egypt, Georgia, Greece, Hungary, Israel, Libya, Morocco, Palestine, Romania, Saudi Arabia, Tunisia, and Türkiye. The final outbreak dataset spanned 2005–2026 and included 744 georeferenced records (Figure 1). Most reports originated from Morocco (39.0%), Algeria (19.0%), and Greece (11.6%), followed by Romania (9.1%), Tunisia (8.9%), and Israel (4.6%), with the remaining records distributed across 10 other countries of the specified domain (Figure 1a). Sheep accounted for the largest share of reported host categories (42.9%), followed by mixed sheep-and-goat herds (29.8%) and goats alone (5.9%); the remaining records involved other mixed domestic livestock groupings (Figure 1b). Reported outbreaks in the merged dataset showed two clear temporal peaks, in 2008 and 2024, with smaller resurgence periods in 2012, 2019, and 2021–2022 (Figure 1c).

### 2.2. Predictor Variables and Raster Preprocessing

Predictive modeling of PPR spatial suitability followed the general framework adapted from Alkhamis et al. [16] and incorporated 25 ecological and anthropogenic predictors selected for their plausible influence on the distribution of susceptible small ruminants and on environmental and anthropogenic conditions shaping contact opportunities and transmission. Because the source datasets were available at different native spatial resolutions, all layers were first cropped to the study extent and then aligned to a common geographic analysis grid prior to model fitting and prediction (Supplementary Table S1). All spatial data were processed in an unprojected geographic coordinate reference system, WGS 84 (EPSG:4326). Continuous raster predictors were first aggregated to the common analysis grid and then aligned by bilinear interpolation, whereas the livestock production-system layer was aligned using nearest-neighbor assignment to preserve class structure. Climatic conditions were represented using WorldClim v2.1 bioclimatic variables derived from long-term temperature and precipitation surfaces, retrieved at 2.5 arc-min native resolution and aligned to the analysis grid. In line with published guidance on the use of quarter-based bioclimatic layers, BIO8, BIO9, BIO18, and BIO19 were excluded to minimize discontinuities from seasonal quarter definition biases [16,31,32]. Elevation (SRTM/WorldClim v2.1) was included as a topographic predictor (native 5 arc-min resolution), and mean wind speed was included as an additional atmospheric covariate available within the WorldClim v2 framework [16,33]. Host availability was quantified using species-specific sheep and goat density layers from Gridded Livestock of the World (GLW3), which provides global livestock distributions for 2010 at 5 arc-min resolution and is widely used in livestock epidemiology [34]. These gridded livestock surfaces are generated by redistributing census counts using spatial covariates, with machine-learning approaches (including Random Forest) documented for improving downscaling performance in this framework [16,31]. To capture heterogeneity in management and mobility contexts, we integrated ruminant production-system information from the FAO Global Livestock Environmental Assessment Model (GLEAM 2018; v3.0; https://www.fao.org/gleam/en/) (accessed on 10 March 2026) framework (e.g., grazing vs. mixed systems; agro-ecological stratification), which operationalizes production systems using biophysical and demographic criteria relevant to animal movement and contact structure [35]. Land cover was characterized using FAO GLC-SHARE (native 30 arc-second resolution), from which proportional coverage layers for key classes (grasslands, croplands, and artificial surfaces) were derived [36]. Human footprint was represented using GPWv4 population density (CIESIN/SEDAC; 30 arc-second grid; 2015/v4.0), providing a standardized measure of population distribution and potential market/reporting interfaces [16,37]. Finally, connectivity was represented using road-infrastructure data from the Global Roads Inventory Project (GRIP4); we represented road density (native 5 arc-min resolution; 2018/v4.0) on the analysis grid as an index of transportation corridors and accessibility that may facilitate animal movement and pathogen spread [16,38]. A complete listing of candidate predictors, source datasets, temporal references, native spatial resolutions, preprocessing steps, and retention status after correlation screening and Boruta selection is provided in Supplementary Table S1. We used a two-step reduction strategy to mitigate multicollinearity and overfitting. First, Pearson correlations were computed across extracted numeric predictor values, and variables were screened using a threshold of |r| > 0.7 (Supplementary Figure S2). Then, Boruta feature selection was applied to identify predictors with robust importance beyond randomized shadow features [19]. Because several predictor layers represented long-term climatic surfaces or otherwise static benchmark datasets, the resulting suitability maps were intended to capture broad regional suitability gradients rather than year-specific outbreak dynamics.

### 2.3. Model Fitting and Spatial Cross-Validation

The models described by Alkhamis et al. [16] were fit based on four classifiers commonly used for spatial disease risk mapping: a binomial generalized linear model (GLM), Random Forest (RF) [20,24], a support vector machine (SVM) [25], and gradient-boosted trees (XGBoost) [26]. Models were trained using the caret framework [39]. For a balanced environmental contrast, presence points were paired with randomly sampled pseudo-absence locations at a 1:1 ratio. Random background sampling was used to represent the full available environmental background within the predefined calibration domain, rather than to define a priori areas as environmentally unsuitable for reported PPR occurence. To assess the sensitivity of model outputs to background prevalence, we also evaluated alternative presence–background ratios of 1:2 and 1:3 during preliminary pipeline testing. These comparisons did not produce meaningful changes in the broad spatial prediction patterns or in rank-based discriminatory performance (AUC). The final analyses, therefore, used a strict 1:1 presence–pseudo-absence design to retain a balanced modeling dataset and a simpler final workflow. After complete-case filtering, the final modeling dataset contained 704 observations, comprising 352 presence locations and 352 pseudo-absence locations. To account for spatial dependence, five-fold spatial blocking was applied using blockCV [27,28]. In the final workflow, block size was not specified manually in cv_spatial(); blockCV therefore determined the partitioning automatically, and the iteration = 50 setting was used during spatial fold allocation to search across alternative random assignments when constructing the final folds. Accordingly, these performance estimates are more informative for geographic generalization under spatial separation than for precise prediction at the fine local scale. Performance was summarized using area under the receiver operating characteristic curve (AUC), sensitivity, specificity, and accuracy from spatial cross-validation, and the Matthews correlation coefficient was computed from pooled cross-validated predictions [40,41,42]. These steps were intended to reduce the influence of clustered reporting and provide a more conservative assessment of geographic transferability under uneven surveillance. For each model, suitability was predicted across the full raster stack and the resulting surface was then clipped to North Africa for interpretation. Agreement across models was used to assess the stability of the broader regional signal, while detailed ecological interpretation was based on the retained model. To quantify spatial consistency, we performed a cell-wise Pearson correlation analysis on the continuous predictive raster surfaces generated by the two top-performing models. Thus, the broader calibration domain informed model fitting and environmental contrast, while the epidemiological interpretation remained centered on North Africa.

### 2.4. Model Interpretability

We assessed the predictors’ influence using several complementary interpretive approaches. Variable-importance measures were first used to compare the broad predictor structure across algorithms. Centered individual conditional expectation (c-ICE) curves were then used to visualize how selected predictors influenced fitted suitability across their observed ranges. Interaction strength was further examined using Friedman’s H-statistics [18,43]. Within the retained RF model, main effects were interpreted from single-predictor response patterns, whereas interactions were interpreted from Friedman’s H-statistics and pairwise partial-dependence plots. Local contributions to predicted suitability were examined using Shapley values for representative high- and low-suitability locations within the iml framework [44]. Detailed ecological interpretation was focused on the retained model.

## 3. Results

**Figure 2:**
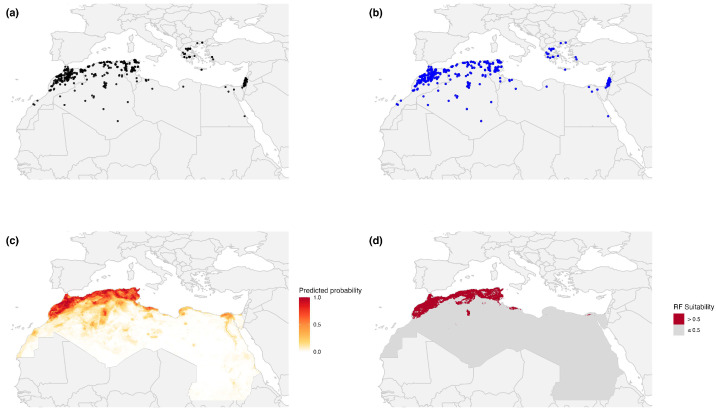
Reported Peste des Petits Ruminants (PPR) outbreaks and Random Forest-predicted suitability in North Africa and the Mediterranean-interface calibration domain, 2005–2026. (**a**) All compiled outbreak reports from the merged FAO EMPRES-i and WOAH WAHIS dataset. (**b**) Spatially thinned outbreak locations retained after 10 km thinning. (**c**) Random Forest-predicted suitability. (**d**) Binary display of Random Forest suitability using a 0.5 visualization threshold.

Reported outbreaks in the merged dataset showed two clear temporal peaks, in 2008 and 2024, with smaller resurgence periods in 2012, 2019, and 2021–2022 (Figure 1c). Across countries, reports were concentrated in Morocco, Algeria, and Greece, followed by Tunisia, Romania, and Israel (Figure 1a). Most host reports involved sheep-related categories, especially domestic sheep and mixed sheep–goat holdings (Figure 1b). To quantify spatial suitability for reported PPR outbreaks under heterogeneous reporting, we fitted four classifiers and evaluated them under five-fold spatial cross-validation (Table 1). After 10 km spatial thinning, 359 outbreak locations were retained (Figure 2b); after raster extraction and complete-case filtering, 352 presence locations remained in the final modeling dataset used for classifier training and evaluation (Table 1). All models showed good discriminatory performance, with AUC values ranging from 0.87 to 0.94. RF and XGBoost achieved the highest AUC values (0.94). Random Forest achieved the highest accuracy (87.93%) and specificity (94.32%), whereas XGBoost achieved the highest sensitivity (82.67%). SVM showed the most even balance between sensitivity (80.97%) and specificity (88.92%), whereas GLM performed less well overall (AUC = 0.87; Table 1). RF and XGBoost achieved the joint-highest AUC. A cell-wise Pearson correlation analysis of their continuous predictive raster surfaces showed strong spatial concordance (Pearson’s r = 0.956). Among these two top-performing models, RF was retained as the primary model for detailed ecological interpretation because it also achieved the highest specificity and overall accuracy under five-fold spatial block cross-validation. The remaining classifiers were used mainly to confirm the stability of the broader regional signal.

The RF suitability surface recapitulated the primary outbreak clusters along the Mediterranean coastline while also identifying additional areas of moderate suitability in settings with relatively low notification density, including parts of central Libya and the eastern Egyptian coastal plain (Figure 2c). When a 0.5 threshold was applied for visualization, most high-suitability cells remained concentrated within the same coastal belt where reported outbreaks were most frequent (Figure 2d). These areas are better understood as places where environmental conditions, host density, and selected human-related factors increase the likelihood that outbreaks will be observed and reported, and thus may warrant closer passive-surveillance attention, rather than as proof of hidden transmission. In that sense, the RF suitability surface is better viewed as a spatial prioritization tool for surveillance and preparedness than as a direct forecast of outbreak occurrence. Suitability remained highest in connected coastal production zones and lower toward the Saharan interior, consistent with lower host density and harsher climatic conditions.

**Figure 3:**
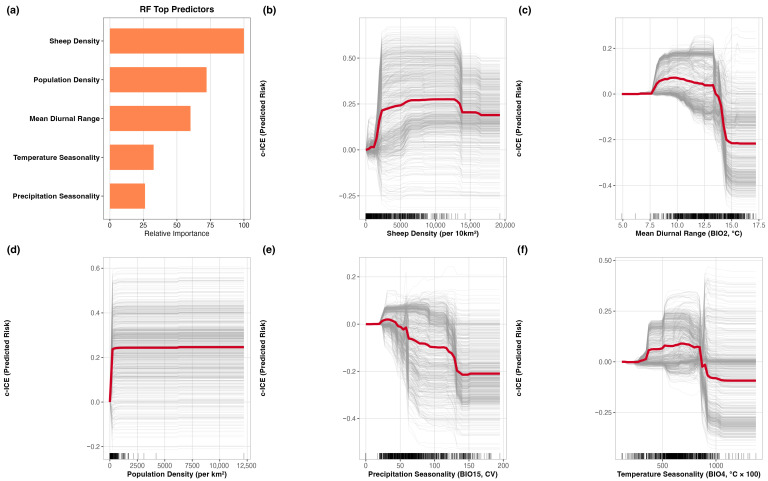
Random Forest feature importance and centered individual conditional expectation (c-ICE) curves for the top five predictors shaping reported Peste des Petits Ruminants (PPR) outbreak suitability in North Africa. (**a**) Relative importance of the top five RF predictors. (**b**–**f**) c-ICE plots for sheep density, mean diurnal range, population density, precipitation seasonality, and temperature seasonality. Thin gray lines represent location-specific responses, whereas the red line represents the partial dependence. Together, these plots highlight non-linear and threshold-like relationships between predicted suitability and key host, climatic, and anthropogenic predictors.

Across algorithms, sheep density was consistently retained among the leading predictors (Figure 3). Because RF was selected as the main model for interpretation, the detailed ecological reading focused on its response patterns. In RF, sheep density emerged as the leading predictor, followed by other host, climatic, and anthropogenic variables, including population density and mean diurnal range. The c-ICE plots showed that the effect of sheep density was not constant across its range. Suitability rose sharply at low sheep densities and plateaued at ~2500–4000 sheep per km^2^. The remaining RF response curves indicated that other leading predictors also had non-linear effects on suitability. Taken together, these results suggest that suitability in North Africa is shaped by threshold-like and non-linear relationships rather than by simple linear gradients.

**Figure 4:**
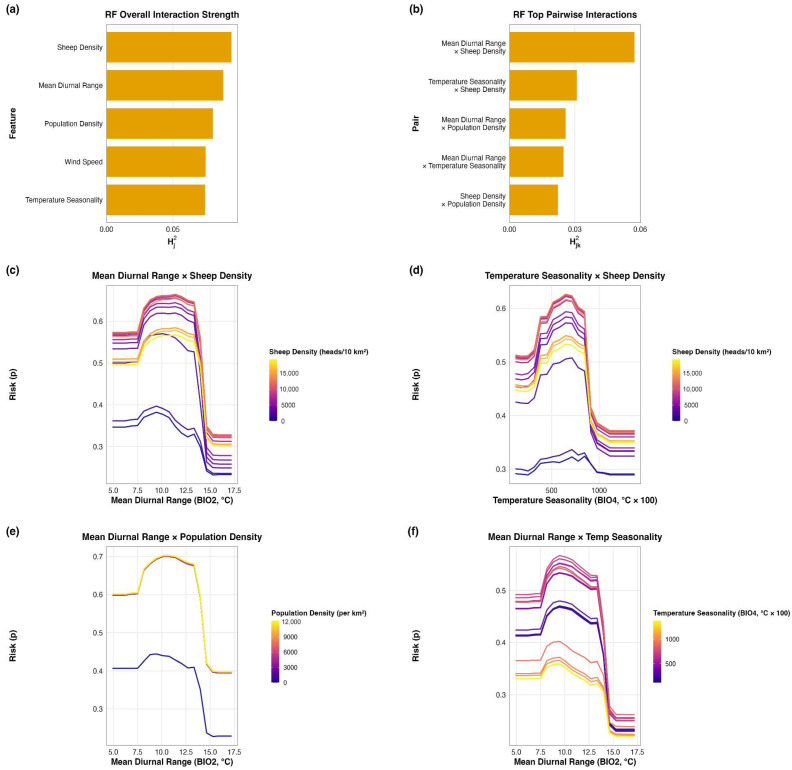
Random Forest interaction diagnostics for leading predictors shaping reported Peste des Petits Ruminants (PPR) outbreak suitability in North Africa. (**a**) Overall interaction strength of the leading RF predictors. (**b**) Top five pairwise interactions ranked by Friedman’s H-statistic. (**c**–**f**) Partial-dependence plots for the four strongest RF interactions. Together, these plots indicate that interactions among predictors contribute meaningfully to the mapped suitability pattern beyond the effects of single predictors alone.

Interaction diagnostics showed that mapped suitability was shaped by joint effects among host, climatic, and anthropogenic predictors rather than by single predictors acting independently (Figure 4). In the RF model, mean diurnal range × sheep density was the strongest pairwise interaction, followed by temperature seasonality and population density interactions. The partial-dependence plots showed that the response to one predictor varied with the level of the other, indicating departures from a simple additive pattern. All together, these results suggest that main effects alone do not adequately explain reported-outbreak suitability in North Africa and that interaction structure contributes significantly to the observed spatial signal. The RF interaction patterns were also consistent with the broader concentration of suitability along the North African coastal belt, especially in coastal Morocco and northern Algeria. Shapley decompositions supported the same general interpretation at the local scale and are provided in Figure S3.

## 4. Discussion

### 4.1. Key Contributions and Main Findings

In this study, outbreak locations represent reported events and therefore reflect not only disease occurrence, but also detectability and reporting opportunity within the surveillance system. The study applied an interpretable ecological niche modeling framework to characterize PPR spatial suitability across North Africa under five-fold spatial block cross-validation. The Mediterranean coastal belt emerged as the main zone of higher reported-outbreak suitability, while lower suitability predominated across much of the Saharan interior. Among the evaluated classifiers, RF showed the strongest overall performance and was therefore retained as the primary model for ecological interpretation. RF-based interpretation indicated that sheep density, population density, mean diurnal range, and seasonal temperature and precipitation metrics were the primary contributors to suitability patterns. Interaction diagnostics further showed that these predictors did not act in isolation, reinforcing the view that the main coastal suitability pattern emerged from combined host, environmental, and anthropogenic conditions rather than from any single variable alone. At the same time, this pattern should be interpreted as identifying settings where reported outbreaks are most likely to be observed under passive surveillance, rather than as a direct representation of the full spatial extent of infection.

### 4.2. Host Availability and Climatic Structure as Determinants of Suitability

Sheep density emerged as the most consistently important predictor, which is biologically plausible given that PPR transmission depends on the availability and mixing of susceptible small ruminants [45]. The c-ICE plots indicated that the relationship between sheep density and predicted suitability was non-linear rather than constant across its range. In the RF model, suitability increased rapidly at lower sheep densities and then changed more gradually once densities reached roughly 2500–4000 sheep/km^2^. Climate variables, particularly mean diurnal range, temperature seasonality, and precipitation seasonality, were also repeatedly retained among the leading predictors, suggesting that suitability reflects not only host availability but also broader environmental gradients associated with reported PPR suitability across North Africa. These variables likely capture ecological gradients that distinguish the more densely connected Mediterranean production belt from the arid interior, where host density, land use, and overall suitability for sustained transmission are less consistently aligned. The additional importance of population density and the interaction structure involving sheep density indicate that the mapped pattern is not explained by climate alone, but by the combined effects of host concentration, environmental seasonality, and anthropogenic context.

### 4.3. Connectivity Patterns and Transmission Opportunity

Interaction diagnostics showed that suitability was shaped by joint rather than isolated effects of host, climatic, and anthropogenic predictors. In the RF model, the leading pairwise interaction involved mean diurnal range and sheep density, indicating that host availability and environmental structure worked together to shape the mapped suitability pattern. This should not be read as evidence that one single interaction universally explains the region. Rather, it suggests that reported-outbreak suitability emerges from combinations of host concentration, environmental seasonality, and anthropogenic connectivity, and that main-effect predictors alone are not sufficient to explain the final spatial signal. At the regional scale, this helps explain why the main suitability signal remained concentrated along the coastal production belt, particularly coastal Morocco and northern Algeria, where livestock density, anthropogenic connectivity, favorable environmental conditions, and reporting opportunity are more likely to overlap. These results suggest that areas where susceptible host populations, favorable environmental conditions, and stronger anthropogenic connectivity coincide, especially along the North African coastal production belt, are likely to be important settings for strengthening passive surveillance, preparedness, and rapid outbreak detection. However, lower reported-outbreak suitability should not be interpreted as settings of low surveillance priority or disease absence. Rather, such areas may warrant targeted active surveillance where transmission is plausible but visibility, access, or reporting sensitivity are limited [4]. In this sense, the maps are best used to support a complementary surveillance strategy rather than a single-priority approach.

### 4.4. Limitations

Despite good discriminatory performance under blocked cross-validation, several limitations should be considered. The spatial blocking scheme was deliberately conservative. Because block size was determined automatically, the resulting validation should be viewed as a test of geographic transferability at the regional scale under spatial separation, rather than as evidence of fine-scale local interpolation accuracy. Using a broader Mediterranean-interface calibration domain improved environmental representation during model fitting, but it also means that the final North Africa suitability surfaces should be interpreted as regionally informed rather than as products of a North Africa-only calibration set. The outbreak notifications compiled from FAO EMPRES-i and WOAH WAHIS reflect heterogeneous surveillance sensitivity and reporting completeness across countries and years. The mapped surfaces should be interpreted as estimates of spatial suitability for reported PPR occurrence, not as calibrated probabilities of infection or outbreak presence. Accordingly, the current predictions should not be interpreted as direct estimates of true disease risk, because uneven surveillance and reporting may still influence where outbreaks are observed and recorded. In practice, the model captures where ecological, host, and anthropogenic conditions were repeatedly compatible with reported events under uneven surveillance, rather than a standardized measure of true infection pressure across the region. Areas of lower suitability may therefore reflect reduced detectability, limited reporting opportunity, or weaker surveillance sensitivity, rather than true absence of disease. In addition, pseudo-absences provide a practical environmental contrast for regional modeling, but they do not represent confirmed disease-free locations and therefore remain dependent on assumptions about where true absences may plausibly occur. Alternative environmental-space pseudo-absence designs may reduce sample-location bias, but in passive outbreak settings they also require stronger assumptions about unsuitable conditions than were warranted by the available data. Most predictors were climatic or otherwise time-invariant, which limits inference on short-term outbreak dynamics driven by seasonal movement, market cycles, and campaign timing. Key intervention variables, such as subnational vaccination coverage, campaign timing, movement restrictions, and surveillance effort, could not be explicitly incorporated. Because these intervention-related variables were not directly modeled, the reported suitability patterns may partly reflect residual confounding from spatial variation in vaccination implementation, movement control, and animal mobility, and should therefore not be interpreted as causal estimates of intervention effects. As a result, the models are better suited to identifying the broad suitability structure than to attributing local variation to specific control failures or intervention gaps. Some anthropogenic predictors, particularly population density and road density, may capture not only movement opportunity and market connectivity, but also variation in outbreak detectability and reporting access across the region. Future work should integrate operational data layers, including vaccination strata, surveillance-effort indicators, reporting-access proxies, and movement data, to improve causal interpretation, reduce reporting-related bias, and better distinguish reported suitability from true disease risk.

## 5. Conclusions

This study applied an interpretable machine-learning ecological niche modeling framework to map the spatial suitability of reported PPR outbreaks across North Africa. The RF model identified the Mediterranean coastal belt as the principal zone of reported-outbreak suitability, with the strongest signal in coastal Morocco and northern Algeria and additional elevated suitability in coastal Tunisia, whereas much of the Saharan interior remained less suitable. Host availability and climatic structure, particularly sheep density, together with diurnal and seasonal temperature and precipitation metrics, were important contributors to the mapped suitability pattern. From a practical standpoint, the maps help identify where passive surveillance is most likely to be effective, particularly along the coastal production belt where livestock concentration, environmental suitability, and anthropogenic connectivity coincide. However, areas with lower reported-outbreak suitability should not be interpreted as free of disease and may require complementary active surveillance to identify transmission that remains unseen or unreported. More broadly, the workflow provides a reproducible and interpretable baseline for integrating outbreak data, environmental predictors, and model explanation in support of PPR preparedness, regional planning, and progressive control efforts in North Africa. It also provides a foundation that can be strengthened in future work through the incorporation of operational data on vaccination, animal movement, and surveillance intensity.

## Figures and Tables

**Table 1:** Cross-validation performance of four machine-learning classifiers for PPR suitability modeling in North Africa.

Final Modeling Dataset: 352 Retained PPR-Positive Locations and 352 Pseudo-Absence Points (n = 704)					
Model	Accuracy	Specificity	Sensitivity	MCC	AUC
RF	87.93	94.32	81.53	0.76	0.94
XGB	87.22	91.76	82.67	0.75	0.94
SVM	84.94	88.92	80.97	0.70	0.92
GLM	81.25	88.35	74.15	0.63	0.87

RF: Random Forest; XGB: Extreme Gradient Boosting; SVM: Support Vector Machine; GLM: Generalized Linear Model; MCC: Matthews correlation coefficient; AUC: area under the receiver operating characteristic curve estimated from five-fold spatial cross-validation.

## Data Availability

The original contributions presented in this study are included in the article and Supplementary Materials. Further inquiries can be directed to the corresponding author. The data presented in this study were derived from publicly available resources, including FAO EMPRES-i, WOAH WAHIS, and publicly available environmental raster datasets. R codes used to generate the results of the present study are available on the following GitHub repository: https://github.com/imanb004/Peste_des_Petits_Ecological_Niche_Modeling-in-North-Africa_Using_Machine_Learning (Accessed on 19 April 2026).

## Supplementary Materials

The following supporting information can be downloaded at: https://www.mdpi.com/article/10.3390/pathogens15050466/s1, Figure S1: Spatial and temporal distribution of Peste des Petits Ruminants (PPR) outbreaks in the Mediterranean region (2005–2026). (a) Temporal distribution of the outbreaks on a biannual basis. H1 and H2 represent 1st and second halves of the corresponding year, respectively. (b) Spatial distribution of the outbreaks, color-coded by database source; Table S1: Candidate predictors and data sources used in the modeling workflow; Figure S2: Correlation coefficient heatmap for features included in all models. Features where |ρ| > 0.7 were already excluded from the dataset; Figure S3: Shapley-value plots showing local feature contributions to predicted reported Peste des Petits Ruminants (PPR) outbreak suitability for representative high- and low-suitability locations. Positive ϕ values indicate that a feature increased local suitability, whereas negative ϕ values indicate that a feature decreased it.

## Abbreviations

The following abbreviations are used in this manuscript:

AUC	Area Under the Receiver Operating Characteristic Curve
CAHFS	Center for Animal Health and Food Safety
c-ICE	centered Individual Conditional Expectation
CIESIN	Center for International Earth Science Information Network
EMPRES-i	Emergency Prevention System for Animal Health Information
EPSG	European Petroleum Survey Group
FAO	Food and Agriculture Organization of the United Nations
GF-TADs	Global Framework for the Progressive Control of Transboundary Animal Diseases
GLEAM	Global Livestock Environmental Assessment Model
GLC-SHARE	Global Land Cover-SHARE
GLM	Generalized Linear Model
GLW3	Gridded Livestock of the World, version 3
GPWv4	Gridded Population of the World, version 4
GRIP4	Global Roads Inventory Project, version 4
ICE	Individual Conditional Expectation
MCC	Matthews Correlation Coefficient
ML	Machine Learning
PPR	Peste des petits ruminants
PPRV	Peste des petits ruminants virus
RF	Random Forest
RNA	Ribonucleic Acid
SEDAC	Socioeconomic Data and Applications Center
SHAP	SHapley Additive exPlanations
SRTM	Shuttle Radar Topography Mission
SVM	Support Vector Machine
TAD	Transboundary Animal Disease
USDA	United States Department of Agriculture
WAHIS	World Animal Health Information System
WGS 84	World Geodetic System 1984
WOAH	World Organisation for Animal Health
XGBoost	Extreme Gradient Boosting
