# Supplementary material for: Spatial Suitability of Peste des Petits Ruminants in North Africa Using Machine-Learning Ecological Niche Modeling
Source: Pathogens. 2026 Apr 24;15(5):466. doi: 10.3390/pathogens15050466 (PMC13209501; doi:10.3390/pathogens15050466)
Supplement: Supplementary file 1 [file pathogens-15-00466-s001.zip › pathogens-4270916-supplementary.pdf]

Spatial Suitability of Peste des Petits Ruminants in North Africa Using Machine-Learning Ecological Niche Modeling

Dinara Imanbayeva [1,*], Moh A. Alkhamis [2], John M. Humphreys[3] and Andres M. Perez [4]

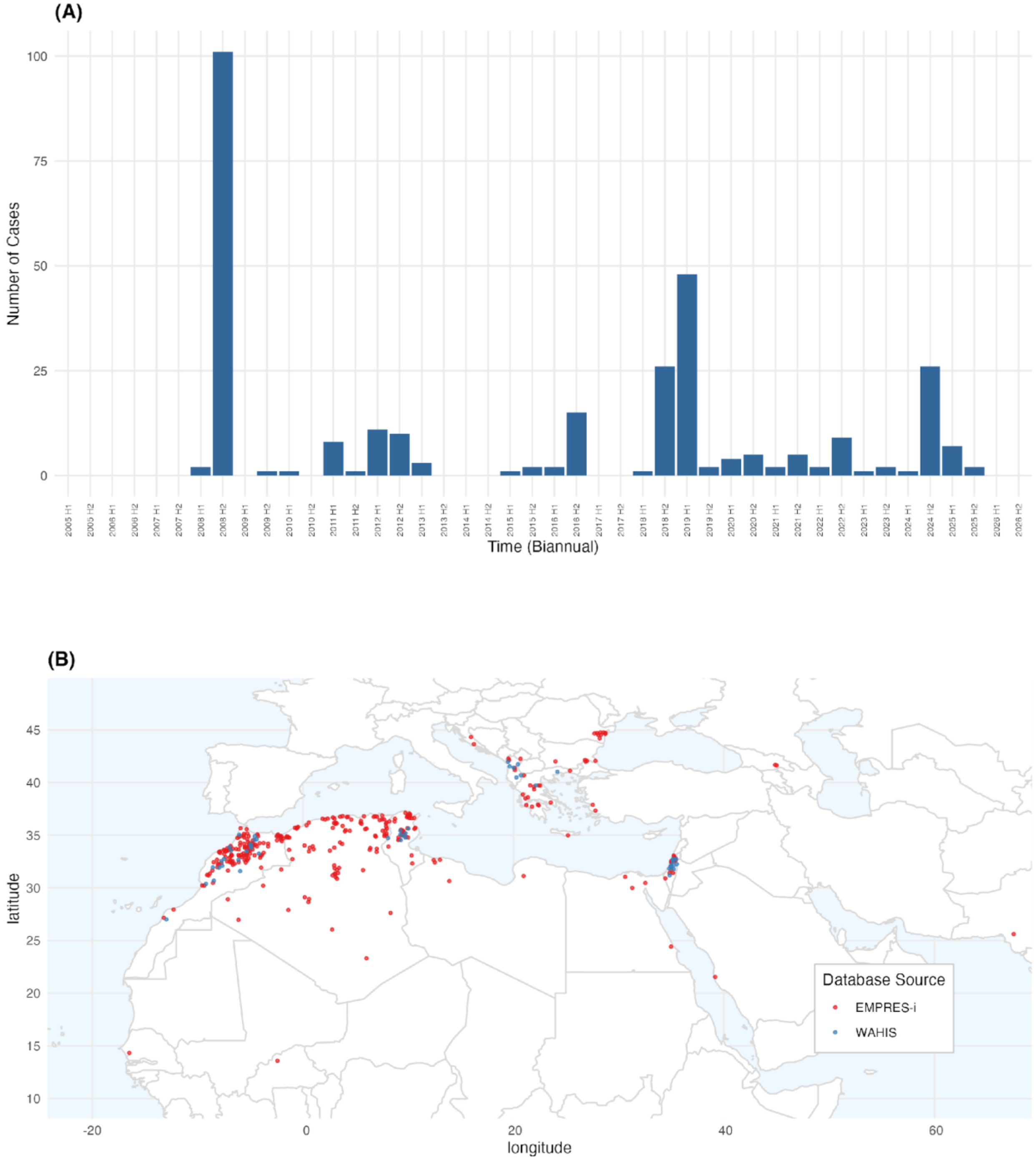

Figure S1. Spatial and temporal distribution of Peste des Petits Ruminants (PPR) outbreaks in the

Mediterranean region (2005–2026). (**a**) Temporal distribution of the outbreaks on a biannual basis. H1 and H2 represent 1st and second halves of the corresponding year, respectively. (**b**) Spatial distribution of the outbreaks, color-coded by database source.

Table S1. Candidate predictors and data sources used in the modeling workflow.

Category	Input Name	Description	Source/Value
Data	All_Mediter_Outbreaks_4_11.csv	PPR Outbreak Locations (Mediterranean Basin)	User Provided
Data	worldregions.shp	Regional Boundary Mask (Filtered to 'Northern Africa')	User Provided (worldregions folder)
Predictor	wc2.1_2.5m_bio_1.tif	Annual Mean Temperature	WorldClim
Predictor	wc2.1_2.5m_bio_2.tif	Mean Diurnal Range	WorldClim
Predictor	wc2.1_2.5m_bio_3.tif	Isothermality	WorldClim
Predictor	wc2.1_2.5m_bio_4.tif	Temperature Seasonality	WorldClim
Predictor	wc2.1_2.5m_bio_5.tif	Maximum Temperature of Warmest Month	WorldClim
Predictor	wc2.1_2.5m_bio_6.tif	Minimum Temperature of Coldest Month	WorldClim
Predictor	wc2.1_2.5m_bio_7.tif	Temperature Annual Range	WorldClim
Predictor	wc2.1_2.5m_bio_10.tif	Mean Temperature of Warmest Quarter	WorldClim
Predictor	wc2.1_2.5m_bio_11.tif	Mean Temperature of Coldest Quarter	WorldClim
Predictor	wc2.1_2.5m_bio_12.tif	Annual Precipitation	WorldClim
Predictor	wc2.1_2.5m_bio_13.tif	Precipitation of Wettest Month	WorldClim
Predictor	wc2.1_2.5m_bio_14.tif	Precipitation of Driest Month	WorldClim
Predictor	wc2.1_2.5m_bio_15.tif	Precipitation Seasonality	WorldClim
Predictor	wc2.1_2.5m_bio_16.tif	Precipitation of Wettest Quarter	WorldClim
Predictor	wc2.1_2.5m_bio_17.tif	Precipitation of Driest Quarter	WorldClim
Predictor	glc_shv10_03.Tif	Grassland Cover	Global Land Cover
Predictor	glc_shv10_02.Tif	Cropland Cover	Global Land Cover
Predictor	glc_shv10_01.Tif	Artificial Surfaces (Urban)	Global Land Cover

Predictor	wc2.1_5m_elev.tif	Elevation	WorldClim/SRTM
Predictor	5_Gt_2010_Da.tif	Goat Density	Gridded Livestock of the World
Predictor	5_Sh_2010_Da.tif	Sheep Density	Gridded Livestock of the World
Predictor	meanwind.tif	Wind Speed	User Provided
Predictor	glps_gleam_61113_10km.tif	Livestock Production Systems	GLEAM
Predictor	pop_dens2015_2.tif	Population Density	WorldPop/User Provided
Parameter	Spatial Thinning	Minimum distance between presence points	10 km
Parameter	Cross-Validation	Spatial Block CV Method	5 Folds (Random Selection)
Parameter	Algorithms	Machine Learning Models	Random Forest; XGBoost; SVM; GLM
Parameter	Evaluation Metrics	Performance Indicators	ROC; Accuracy; Specificity; Sensitivity; MCC; sAUC

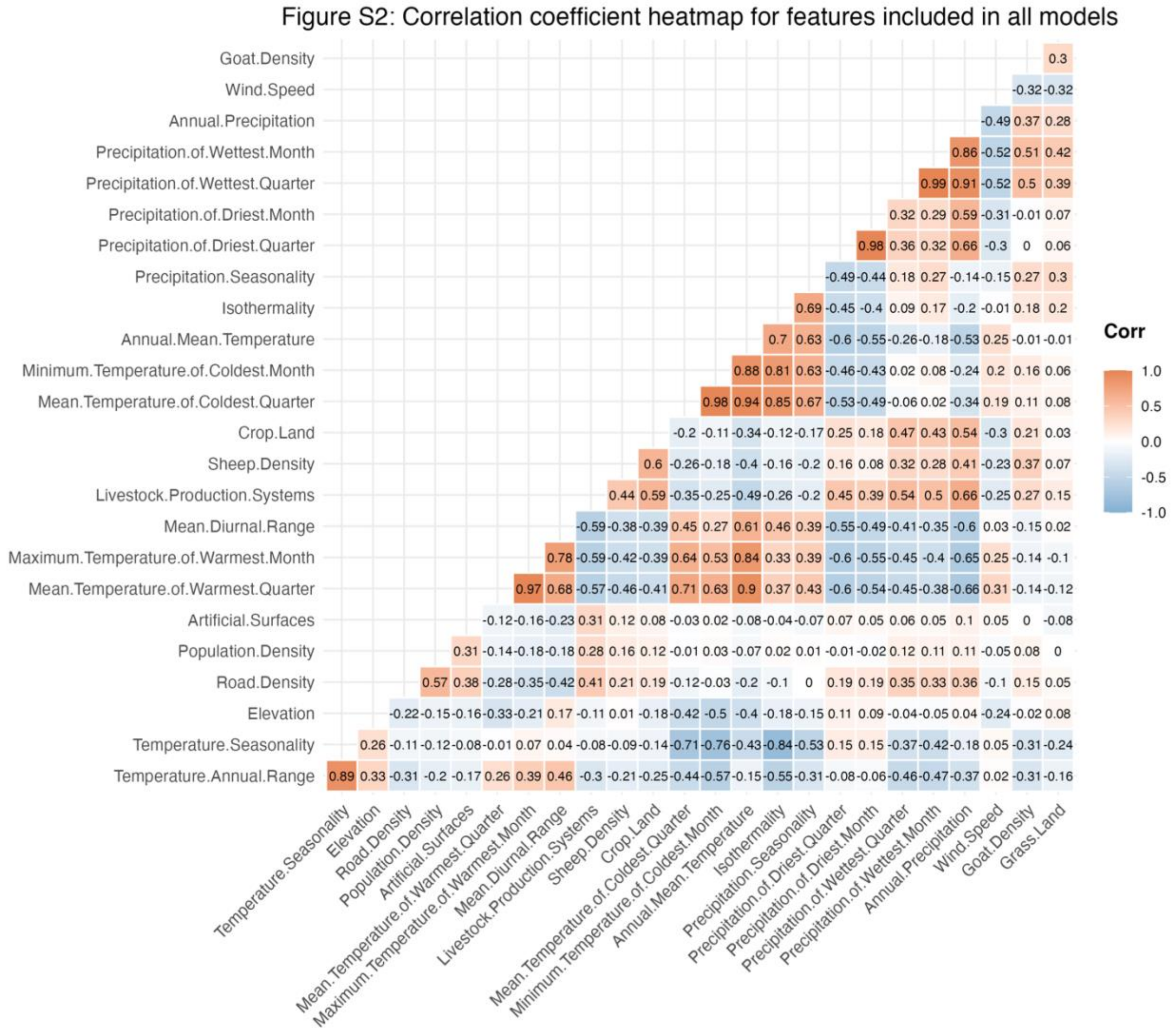

Figure S2. Correlation coefficient heatmap for features included in all models. Features where |ρ| > 0.7 were already excluded from the dataset.

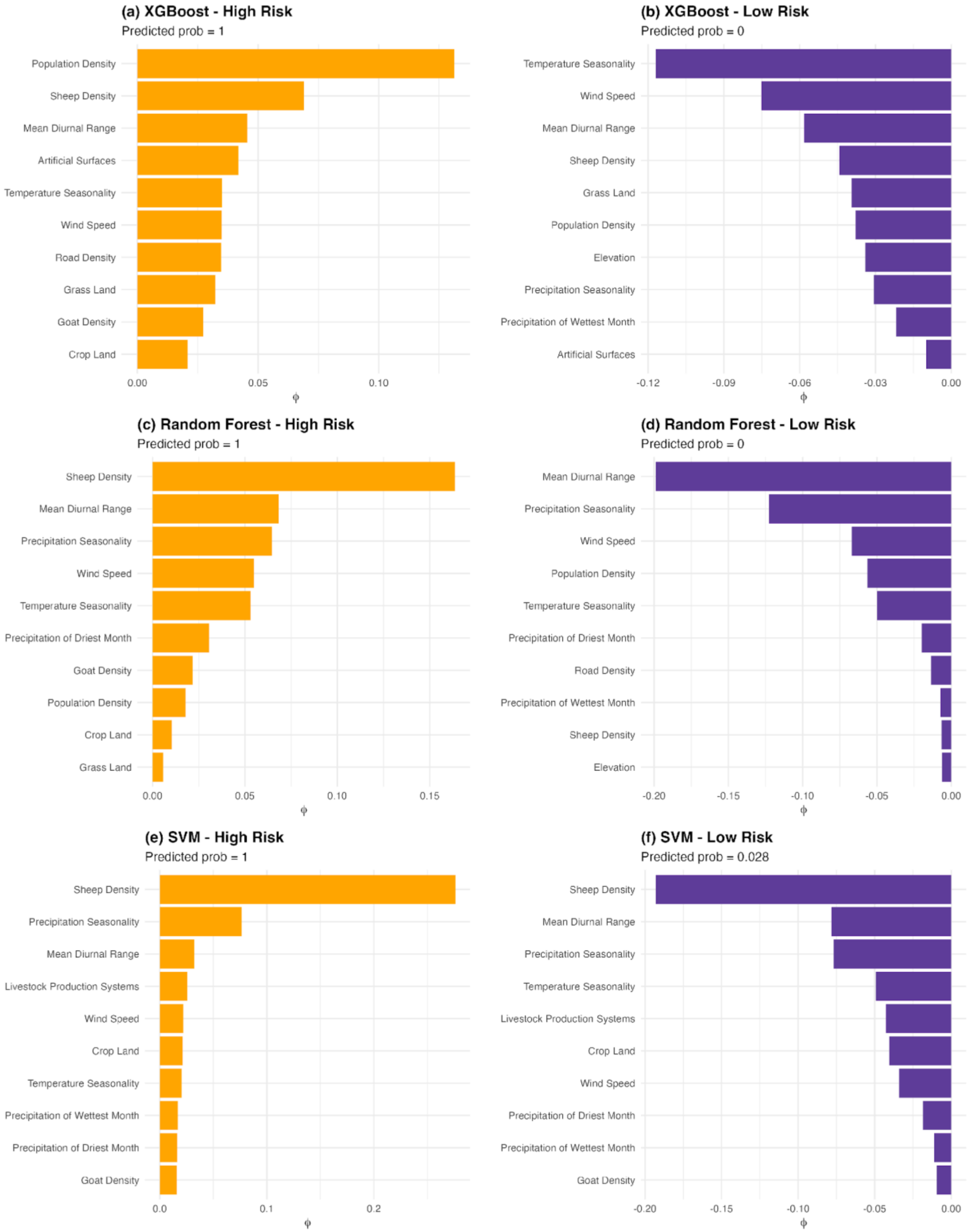

Figure S3. Shapley-value plots showing local feature contributions to predicted reported Peste des Petits Ruminants (PPR) outbreak suitability for representative high- and low-suitability locations. Positive φ

values indicate that a feature increased local suitability, whereas negative φ values indicate that a feature decreased it.
